# Medical Jurisprudence

**Published:** 1850-11

**Authors:** 


### ARTICLE I.

*Medical Jurisprudence.*---By ALFRED TAYLOR, F. R. S., Licentiate of the Royal College of Physicians; Member of the Royal College of Surgeons; and Professor of Medical Jurisprudence and Chemistry at Guy's Hospital. Second American, from the third London edition; with notes and additions by R. EGGLESFIELD GRIFFITH, M. D., &c. Philadelphia: Lea & Blanchard, 1850; pp., 670; 8vo. (From the Publishers, and for sale by Jos. Keen, Jr., & Brother, 161 Lake street, Chicago.)

Medical Jurisprudence is the common ground on which the legal and medical professions meet; although we are sorry to say that it is a soil not sufficiently cultivated by either. We are pleased to see, however, that the apathy heretofore prevailing on this subject is giving way to a more cultivated and enlightened sentiment. As an evidence of this we cite the fact of a demand for a second edition of the work above mentioned. We believe it is universally conceded that a medical man is the proper person to write a work on Medical Jurisprudence, inasmuch as a physician is more conversant with the details of Anatomy, Physiology, &c., than a lawyer can possibly be.

It is not excess of praise to say that the volume before us, is the very best treatise extant on Medical Jurisprudence. In saying this, we do not wish to be understood as detracting from the merits of the excellent works of Beck, Ryan, Traill, Guy and others; but in interest and value we think it must be conceded that Taylor's is superior to anything that has preceded it.

The author is already well known to the profession by his valuable treatise on Poisons; and the present volume will add materially to his high reputation for accurate and extensive knowledge and discriminating judgment. Were we disposed to be hyper-critical, we could lay our finger here and there on a trifling inaccuracy, or an indefiniteness in the employment of technical terms and phrases; but these are comparatively insignificant matters, which do not affect the general value of the volume.

The principles of law applicable to a given case are succinctly but comprehensively announced; the facts material to the general issue are acutely discriminated from those which are unimportant or irrelevant; whilst the value of the volume is much enhanced by details of cases already adjudicated. Dr. Griffith has, in his notes, added many matters of interest with reference to American Statute Law, &c., so that the work is brought completely up to the wants of the physician and lawyer at the present day. M.

